# Diseases of the Stomach and Intestines

**Published:** 1904-05-21

**Authors:** 


### DISEASES OF THE STOMACH AND INTESTINES.

*(Concluded from page 119.)*

Dysentery.—Not only are there two distinct forms of this disease—the bacillary and the amœbic, but each of these is capable of subdivision. Vincent[15] describes three kinds of intestinal amœbæ—the non-pathogenic, the true dysentery amœba pathogenic to man and possibly to the cat and dog, and the *amœba coli mitis* pathogenic to man alone. Broido[16] states that bacillary dysentery may be due to the balantidium coli or to no fewer than four distinct varieties of bacillus. The disease is in fact a group presenting very similar symptoms but owning very various causes. The clumping reaction in cases of Shiga's or Flexner's bacillus is said by Pilsbury[17] to be reliable in children under one year in dilutions of 1 in 20 and in older persons in 1 in 50, though occasionally non-dysenteric adult serum gives an agglutination with a dilution of 1 in 100. Shiga's bacillus clumps rather more readily than Flexner's. Todd[18] has succeeded in preparing an immune horse serum which has high protective power for rabbits; it agglutinates Flexner's bacilli though it has no protective power against injections of the living organism. It is thus a true antitoxin. The toxin was prepared by growing the bacillus in a highly alkaline broth and some little time was necessary for its neutralisation *in vitro*.

Colitis.—Acute ulcerative colitis is a rare and almost necessarily fatal condition. A case with notes of the autopsy has been recorded by Syers[19] occurring in a woman aged 29. There was no obvious cause for the attack which was fatal by perforation and peritonitis. Syers remarks that the latter condition gave rise to no notable symptoms owing to the previously collapsed condition of the patient; while the view that operation after perforation was impracticable was amply borne out by the post-mortem findings, when the whole of the colon was seen to be in a condition of extreme ulceration. The only drug of any service was nitrate of silver by the mouth and as an injection, and its good effect was but slight and transitory. The *bacterium coli commune* has been accused of producing the condition, but there is no real evidence of this, and Strasburger[20] has shown that under normal conditions this organism and the *bacillus lactis aërogenes* prevent fermentation in the small intestine going on to putrefaction and are thus useful symbiontes. In excessive numbers, however, they may use up too much nitrogenous matter and give rise to absorbable toxins.

**Intestinal Atony** on the other hand is a fairly common condition for the relief of which salicylate of physostigmine has recently been proposed by Carlo.[21] It may be administered in a pill, the maximum dose being $\frac{1}{6}$ to $\frac{1}{10}$ grain daily. It stimulates peristalsis and is very useful in meteorism but contra-indicated in inflammatory conditions, such as muco-membranous colitis. In the latter condition Austin[22] has described a method for determining the amount of indol in the fæces as an index of putrefactive changes. He finds it much increased in this disease. Skatol was, however, included in the estimation.

Intestinal Worms.—Since the return of the United States Army from the Philippines, a number of observations have been made on an internal parasite found in many instances which was at first thought to be the ordinary anchylostoma or uncinaria duodenalis, but which has been shown by Stiles to be a distinct form named by him U. Americana. Garrison, Ransom, and Stevenson,[23] who investigated 500 cases of insane patients in hospital, found 13·2 per cent. infected with parasites, and 3 per cent. with U. Americana or duodenalis, the largest number of infections occurring in soldiers returned from the Philippines. Smith,[24] however, as a result of the examination of medical students in Galveston, Texas, considers that U. Americana is indigenous in the States round the Gulf of Mexico, especially where the soil is sandy and the drinking water derived from surface wells. Craig[25] has reported the clinical histories of 12 cases, soldiers from the Philippines, with one death and autopsy. The clinical symptoms are anæmia, distension, and tenderness of the abdomen, with various digestive disturbances, headache, lassitude, and muscular weakness, with dyspnœa on exertion and a weak, irregular pulse. The "cadaveric stare" when the eyes are fixed for a little time on the observer's pupils, as described by Stiles, was frequently found. Both Smith and Craig attach importance to eosinophilia, which, however, is found in all animal parasitic infections of the intestine. Dirt eating was not noted as a cause by either observer, and though both admit that the parasite may enter by skin lesions such as "ground itch," only one case is reported where this was probable, the larva entering coincidently with an attack by the chigoe (*pulex penetrans*). There is general agreement as to the value of thymol taken on an empty stomach and followed by a purge.

15 Lancet, Jan. 30, 1904, p. 317. 16 Ibid. 17 Med. Rec., Dec. 12, 1903, p. 948. 18 Brit. Med. Jour., Dec. 5, 1903, p. 1456. 19 Lancet, Nov. 28, 1903, p. 1500. 20 Med. Rec., Jan. 23, 1904, p. 151. 21 Am. Jour. Med. Sci., Jan., 1903, p. 163. 22 Med. Rec., Dec. 26, 1903, p. 1024. 25 Am. Jour. Med. Sci. 24 Ibid., Nov., 1904, p. 769. 23 Ibid., p. 798.

